# Recent Advances in Transcription Factor–Mediated Regulation of Salvianolic Acid Biosynthesis in *Salvia miltiorrhiza*

**DOI:** 10.3390/plants15020263

**Published:** 2026-01-15

**Authors:** Song Chen, Fang Peng, Shan Tao, Xiufu Wan, Hailang Liao, Peiyuan Wang, Can Yuan, Changqing Mao, Xinyi Zhao, Chao Zhang, Bing He, Mingzhi Zhong

**Affiliations:** 1 College of Life Science, Sichuan Normal University, Chengdu 610101, China; chensong0810@gmail.com (S.C.); peiyuan_w@163.com (P.W.); 2 Industrial Crop Research Institute, Sichuan Academy of Agricultural Sciences, Chengdu 610300, China; pengfang1134@scsaas.cn (F.P.); jzstaoshan@163.com (S.T.); liaohailang1221@scsaas.cn (H.L.); scnkyjzsxy@163.com (C.Y.); mcq1937@163.com (C.M.); zhaoxinyi22@scsaas.cn (X.Z.); 3 State Key Laboratory for Quality Ensurance and Sustainable Use of Dao-di Herbs, Beijing 100700, China; xiufuwan@163.com

**Keywords:** *Salvia miltiorrhiza*, stress response, regulatory network, transcriptional regulation, secondary metabolism

## Abstract

*Salvia miltiorrhiza* Bunge is a traditional Chinese medicinal plant whose roots are rich in water-soluble phenolic acids. Rosmarinic acid and salvianolic acid B are representative components that confer antibacterial, antioxidant, and cardio-cerebrovascular protective activities. However, these metabolites often accumulate at low and unstable levels in planta, which limits their efficient development and use. This review summarises recent advances in understanding salvianolic acid biosynthesis and its transcriptional regulation in *S. miltiorrhiza*. Current evidence supports a coordinated pathway composed of the phenylpropanoid route and a tyrosine-derived branch, which converge to generate rosmarinic acid and subsequently more complex derivatives through oxidative coupling reactions. Key findings on transcription factor families that fine-tune pathway flux by regulating core structural genes are synthesised. Representative positive regulators such as *SmMYB111*, *SmMYC2*, and *SmTGA2* activate key nodes (e.g., *PAL*, *TAT*/*HPPR*, *RAS*, and *CYP98A14*) to promote phenolic acid accumulation. Conversely, negative regulators such as *SmMYB4* and *SmMYB39* repress pathway genes and/or interfere with activator complexes. Major regulatory features include hormone-inducible signalling, cooperative regulation through transcription factor complexes, and emerging post-transcriptional and post-translational controls. Future directions and challenges are discussed, including overcoming regulatory redundancy and strong spatiotemporal specificity of transcriptional control. Integrating spatial and single-cell omics with functional genomics (e.g., genome editing and rational TF stacking) is highlighted as a promising strategy to enable predictive metabolic engineering for the stable, high-yield production of salvianolic acid-type compounds.

## 1. Introduction

*Salvia miltiorrhiza* Bunge (Danshen) is a perennial herb of the Lamiaceae family whose dried roots and rhizomes are widely used in traditional Chinese medicine to promote blood circulation, resolve blood stasis, and alleviate pain and restlessness [1,2]. Phytochemical studies have identified more than 100 constituents from *S. miltiorrhiza* [3]. The pharmacological activities of Danshen are mainly attributed to two major groups of specialised metabolites: lipophilic diterpenoids and water-soluble phenolic acids, among which rosmarinic acid (RA) and salvianolic acid B (Sal B) are the most representative compounds [4,5]. These phenolic acids, collectively referred to as salvianolic acids, exhibit antibacterial, antioxidant, antiviral, and cardio-cerebrovascular protective effects and are widely used for the treatment of vascular disorders such as atherosclerosis and myocardial ischaemia, conferring high medicinal and economic value [6,7]. Nevertheless, salvianolic acids typically accumulate at relatively low levels in planta, and their production is tightly controlled by multiple internal and environmental factors, which hampers the stable supply required for efficient exploitation of these bioactive ingredients. Therefore, a comprehensive understanding of the biosynthetic pathways and regulatory mechanisms underlying salvianolic acid formation is critical for the rational improvement, development, and utilisation of these valuable metabolites.

With the publication of the *S. miltiorrhiza* genome and the rapid accumulation of multi-omics datasets, substantial progress has been made in elucidating the biosynthetic pathway of salvianolic acids and in identifying key structural genes, including phenylalanine ammonia-lyase (PAL), cinnamate 4-hydroxylase (C4H), 4-coumarate:CoA ligase (4CL), tyrosine aminotransferase (TAT), and 4-hydroxyphenylpyruvate reductase (HPPR). These efforts have largely clarified the overall architecture of the phenylpropanoid and tyrosine-derived branches. In parallel, several transcription factor (TF) families, such as MYB, basic helix–loop–helix (bHLH), APETALA2/ethylene-responsive factor (AP2/ERF), and WRKY, have been shown to act as positive or negative regulators of salvianolic acid biosynthesis by modulating the transcription of pathway genes, thereby altering salvianolic acid accumulation. Collectively, these findings outline an emerging regulatory cascade connecting external and endogenous cues with TFs, structural genes, and ultimately metabolite production.

In this review, recent progress in TF–mediated regulation of salvianolic acid biosynthesis in *S. miltiorrhiza* is summarised, with emphasis on the functional features, regulatory mechanisms, and network interactions of different TF families. The aim is to provide a conceptual framework and reference for further dissection of the regulatory networks controlling salvianolic acid metabolism and for the rational improvement and efficient utilisation of these valuable metabolites.

**Figure 1:**
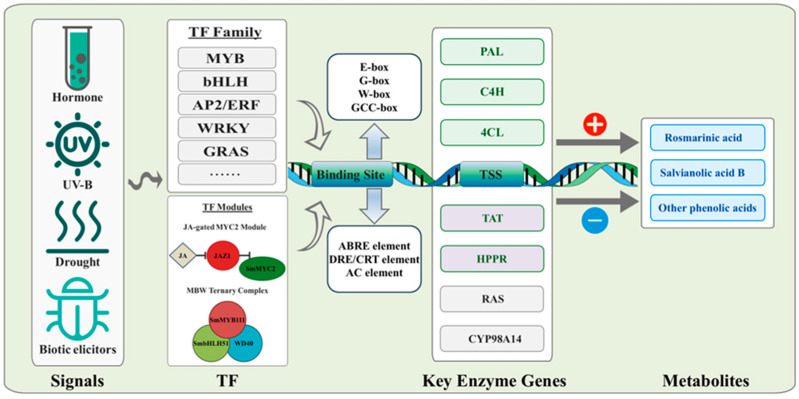
Integrated regulatory framework for salvianolic acid biosynthesis in *S. miltiorrhiza*.

Based on the available evidence, an integrated model linking signals, regulatory modules, promoter cis-elements, key enzyme genes, and metabolite outputs is proposed (Figure 1). This model provides a conceptual framework for the family-by-family discussion below.

## 2. Literature Search Strategy

To ensure a comprehensive and reproducible overview of recent advances, a systematic literature search was conducted following the PRISMA 2020 guidelines [8]. The primary objective was to identify all relevant studies investigating transcription factor-mediated regulation of salvianolic acid biosynthesis in *S*. *miltiorrhiza*.

Electronic searches were performed in the Web of Science Core Collection, PubMed, and the China National Knowledge Infrastructure (CNKI) databases, covering the period from January 2000 up to the date of the search (December 2025). The search strategy combined keywords and Boolean operators: (“*Salvia miltiorrhiza*” OR “*S. miltiorrhiza*” OR Danshen) AND (“salvianolic acid” OR “rosmarinic acid” OR “phenolic acid”) AND (“transcription factor” OR “transcriptional regulat*” OR MYB OR bHLH OR WRKY OR ERF OR NAC). The search was limited to studies published in English or Chinese.

The retrieved records were screened in two stages based on predefined criteria. Inclusion criteria were: (1) original research or review articles focusing on the identification, characterisation, or functional analysis of transcription factors in *S. miltiorrhiza*; (2) studies explicitly linking TF function to the salvianolic acid/rosmarinic acid biosynthetic pathway; (3) studies providing molecular evidence (e.g., gene expression, promoter binding, transgenic validation). Exclusion criteria were: (1) studies focusing solely on chemical profiling, pharmacological effects, or other metabolic pathways (e.g., tanshinones) without mechanistic regulatory insights; (2) conference abstracts, or non-peer-reviewed articles without sufficient experimental detail.

**Figure 2:**
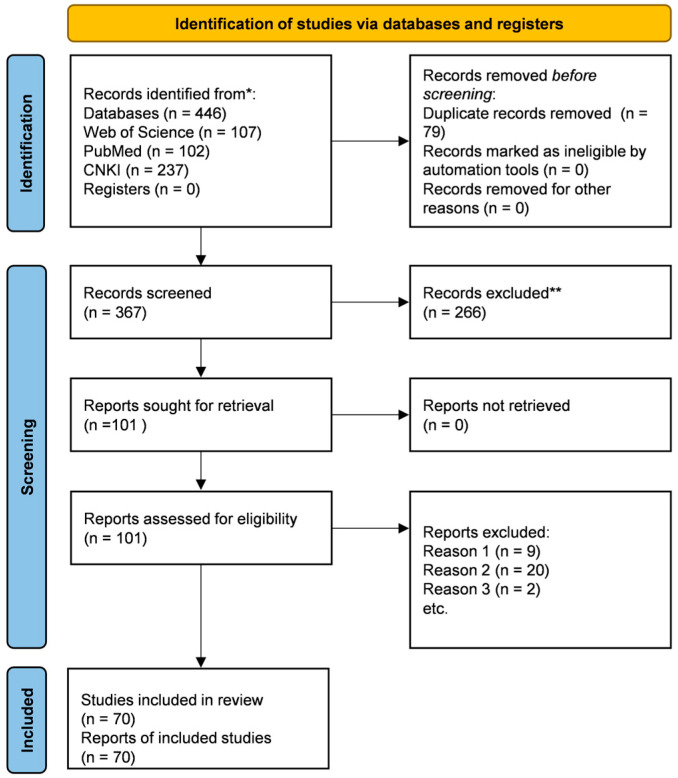
PRISMA flow diagram of the literature search and selection process for studies on transcription factor-mediated regulation of salvianolic acid biosynthesis in *S*. *miltiorrhiza*. **Notes:** * The number of records identified is reported for each database/register searched. ** No automation tools were used; all records were screened and excluded by the reviewers.

The screening process is summarised in the PRISMA flow diagram (Figure 2). A total of 446 records were identified through database searching (107 from Web of Science, 102 from PubMed, 237 from CNKI). After removing 79 duplicates, 367 unique records were screened by title and abstract. Of these, 266 records were excluded, and 101 full-text articles were assessed for eligibility. After full-text review, 31 articles were excluded for the following reasons: (1) duplicate or overlapping study (where a more comprehensive report was available); (2) the studied regulator did not meet our core criteria for an *S. miltiorrhiza* transcription factor (e.g., non-TF proteins or heterologous TFs); or (3) insufficient mechanistic evidence to confirm a direct regulatory function. Consequently, 70 studies were included for qualitative synthesis in this review. Data regarding TF names, families, functions, regulatory mechanisms, and experimental evidence were extracted and thematically organised to construct the current synthesis.

## 3. Biosynthetic Pathways of Salvianolic Acids and Transcriptional Regulation

### 3.1. Salvianolic Acid Biosynthetic Pathways

**Figure 3:**
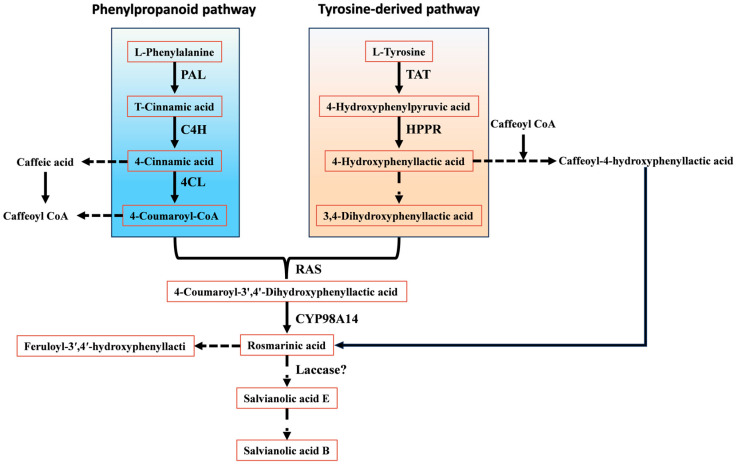
Biosynthetic pathways of salvianolic acids.

Recent work indicates that salvianolic acid biosynthesis in *S. miltiorrhiza* is orchestrated through coordinated operation of the phenylpropanoid pathway and a tyrosine-derived branch [9]. The phenylpropanoid route provides 4-coumaroyl-CoA, whereas the tyrosine-derived route generates 4-hydroxyphenyllactic acid; together, these compounds act as central precursors for RA formation. In the phenylpropanoid pathway, phenylalanine is first deaminated to cinnamic acid by PAL. Cinnamic acid is then hydroxylated and activated to 4-coumaroyl-CoA by C4H and 4CL, respectively. In parallel, tyrosine is converted to 4-hydroxyphenyllactic acid through successive reactions catalysed by TAT and HPPR. These two branches ultimately converge when rosmarinic acid synthase (RAS) and the cytochrome P450 monooxygenase CYP98A14 catalyse condensation of 4-coumaroyl-CoA and 4-hydroxyphenyllactic acid to yield RA, a pivotal intermediate in salvianolic acid biosynthesis (Figure 3).

Notably, Di et al. [10] proposed an alternative major route for RA formation. In this phenylpropanoid branch, 4-coumaric acid is first hydroxylated to caffeic acid (CA) rather than being directly activated to a CoA thioester; CA is subsequently converted to caffeoyl-CoA. In *S. miltiorrhiza*, RAS then catalyses coupling of caffeoyl-CoA with 4-hydroxyphenyllactic acid to produce caffeoyl-4-hydroxyphenyllactic acid, which is finally converted to RA by CYP98A14.

RA serves not only as an end product in *S. miltiorrhiza* but also as a common precursor for most salvianolic acids. On the RA scaffold, further oxidative coupling and polymerisation reactions give rise to structurally more complex phenolic acids, such as salvianolic acid A (Sal A) and Sal B. Current evidence suggests that Sal B biosynthesis proceeds through phenoxy radical–mediated coupling between RA and its derivatives, although the complete set of catalytic enzymes has not yet been comprehensively identified. Structurally, Sal B can be regarded as a condensation product of two CA moieties and two molecules of danshensu, or alternatively as a dimerisation product derived from RA–based phenoxy radicals. Li et al. [11,12] proposed that Sal B may be generated from two RA molecules via radical formation at the 2-position of the aromatic ring and the corresponding α-carbon, followed by radical coupling to yield Sal B directly, or through salvianolic acid E as an intermediate under the action of laccases and other oxidative enzymes. In addition, Xu et al. [13] reconstructed a de novo RA biosynthetic pathway in *Saccharomyces cerevisiae* by heterologous expression of PAL, C4H, 4CL, TAT, HPPR, RAS, and CYP98A14. Interestingly, low levels of Sal B were detected in the engineered yeast even without introduction of a specific polymerase, suggesting that RA may undergo spontaneous oxidative coupling under certain conditions.

Most key structural genes required for salvianolic acid biosynthesis, including *PAL*, *C4H*, *4CL*, *TAT*, *HPPR*, *RAS*, and *CYP98A14*, have been cloned and functionally characterised. Among these enzymes, PAL in the phenylpropanoid pathway is widely regarded as the first rate-limiting step, and its transcript abundance is often positively associated with accumulation of downstream phenolic compounds [14]. TAT and HPPR play critical regulatory roles in the tyrosine-derived branch, and HPPR in particular is considered a major bottleneck enzyme that determines the supply capacity of 4-hydroxyphenyllactic acid [15,16].

### 3.2. Transcription Factors Regulating Salvianolic Acid Biosynthesis

TFs occupy central positions in the regulatory network controlling salvianolic acid biosynthesis and fine-tune pathway output through three principal modes. First, salvianolic acid formation depends on multiple interconnected metabolic routes, including the phenylpropanoid pathway, terpenoid biosynthesis, and polyphenol metabolism. TFs modulate metabolic flux by recognising and binding cis-regulatory elements in the promoters of key structural genes such as *PAL*, *C4H*, *TAT*, *HPPR*, and *CYP98A14*, thereby altering their transcriptional activity [17]. For example, several MYB TFs, including *SmMYB4*, repress expression of *SmPAL*, *SmC4H*, *Sm4CL2*, and *SmTAT*, which results in reduced salvianolic acid accumulation [18], whereas others such as *SmMYB1* act as positive regulators that activate these pathway genes and enhance metabolite production [19]. Second, certain TFs control secondary metabolism by responding to external or endogenous signals; for instance, *SmERF1L1* is induced by jasmonic acid (JA) and markedly decreases the levels of RA and salvianolic acid A. Third, different classes of TFs, including MYB, bHLH, WRKY, and NAC proteins, frequently engage in protein–protein interactions to assemble regulatory complexes that coordinately regulate target gene expression, thereby forming a complex transcriptional network. Within the bHLH family, the core regulator *SmMYC2* has been shown to interact with *SmMYB111*, which attenuates transcriptional activation of key genes in the phenolic acid biosynthetic pathway [20].

To date, members of multiple TF families have been identified as positive or negative regulators of salvianolic acid biosynthesis. Systematic synthesis of their regulatory mechanisms will help refine current models of transcriptional control over salvianolic acid production.

## 4. Characteristics of Transcription Factors Regulating Salvianolic Acid Biosynthesis

### 4.1. Transcription Factor Families and Structural Features

TFs are key components of regulatory networks that control plant secondary metabolism. By recognising specific cis-acting elements in the promoters of target genes, they modulate transcriptional activity and thereby affect the synthesis and accumulation of specialised metabolites. In *S. miltiorrhiza*, several TF families have been associated with salvianolic acid metabolism, including AP2/ERF, bHLH, R2R3-MYB, WRKY, GRAS, basic leucine zipper (bZIP), NAC, TCP, SQUAMOSA promoter-binding protein-like (SPL), and LATERAL ORGAN BOUNDARIES DOMAIN (LBD) proteins.

The MYB family is the largest TF family in plants and has roles in disease resistance, nutrient uptake, responses to environmental stresses, plant development, and regulation of secondary metabolite biosynthesis, such as flavonoids and terpenoids [21]. A defining feature of MYB proteins is the presence of one or more MYB DNA-binding domains, each consisting of an imperfect repeat (R) of approximately 52 amino acids that forms a helix–turn–helix structure [22]. This structure enables specific recognition of MYB core motifs and AC-rich cis-acting elements in target promoters [23]. Based on the number of repeats within the MYB domain, plant MYB proteins are classified into four groups: 1R-MYB, R2R3-MYB, 3R-MYB, and 4R-MYB, among which R2R3-MYB proteins represent the most abundant subtype [24]. In *S. miltiorrhiza*, 110 R2R3-MYB transcription factors have been identified [25]. Most MYB regulators functionally implicated in specialised metabolism (including phenolic acids and tanshinones) reported to date belong to the R2R3-MYB subgroup.

The bHLH family is the second largest TF family in plants after MYB. bHLH proteins share a conserved domain of approximately 60 amino acids, comprising an N-terminal basic region of 15–20 residues responsible for DNA recognition and a C-terminal helix–loop–helix region of 40–50 residues that mediates protein dimerisation. These TFs preferentially bind E-box elements (CANNTG), with the canonical G-box motif (CACGTG) as a common target [26,27]. Based on conserved structural features and phylogenetic relationships, 127 bHLH members have been identified in *S. miltiorrhiza* and assigned to 25 subfamilies [28].

AP2/ERF TFs are plant-specific and are characterised by the presence of a signature AP2 DNA-binding domain and responsiveness to ethylene signalling. The AP2 domain comprises approximately 60–70 amino acids and adopts a typical three-dimensional structure consisting of one α-helix and three β-sheets, which enables recognition of GCC-box (AGCCGCC) elements in target promoters [29]. According to the number and structural features of AP2 domains, the AP2/ERF superfamily is commonly divided into four subfamilies: AP2, ERF/DREB, related to RAV, and Soloist. The ERF/DREB subfamily contains a single AP2 domain and can be further subdivided into the ERF and DREB groups, which are mainly distinguished by the amino acid residues at positions 14 and 19 within this domain [30]. In most plant species, the AP2/ERF family includes more than 100 members, with ERF and DREB constituting the largest groups, followed by AP2 [31]. In *S. miltiorrhiza*, 170 AP2/ERF-type TFs have been identified, and a subset is predicted to regulate salvianolic acid biosynthesis [32].

The WRKY family is named after the conserved N-terminal heptapeptide sequence WRKYGQK within the WRKY domain. WRKY proteins typically contain one or two WRKY domains together with a C2H2- or C2HC-type zinc-finger motif, which enables specific binding to W-box elements ((T)TGAC(C/T)) in target gene promoters [33]. Based on the number of WRKY domains and the type of zinc finger, WRKY TFs are classified into three major groups: group I proteins possess two WRKY domains and a C2H2 zinc-finger motif; group II proteins contain a single WRKY domain and a C2H2 zinc-finger motif; and group III proteins harbour one WRKY domain and a C2HC zinc-finger motif [34]. In *S. miltiorrhiza*, 69 WRKY family members have been identified to date [35].

The GRAS TF family is named after its initially characterised members GIBBERELLIN INSENSITIVE (GAI), REPRESSOR OF ga1-3 (RGA), and SCARECROW (SCR). GRAS proteins generally comprise 400–770 amino acids and contain a highly conserved C-terminal region with five characteristic motifs: leucine heptad repeat I (LHR I), the VHIID core motif, leucine heptad repeat II (LHR II), and the PFYRE and SAW motifs. In contrast, the N-terminal region is highly variable, except in the DELLA subfamily, and often includes intrinsically disordered regions associated with regulatory functions [36,37]. Based on sequence similarity and phylogenetic relationships, the GRAS family is divided into several subfamilies, including DELLA, SHORT-ROOT (SHR), SCR, and PHYTOCHROME A SIGNAL TRANSDUCTION 1 (PAT1). In *S. miltiorrhiza*, 34 GRAS family members have been identified [38].

bZIP TFs represent another family that regulates salvianolic acid biosynthesis. They are characterised by a conserved bZIP domain comprising a basic region responsible for nuclear localisation and DNA binding, and a leucine zipper region formed by heptad repeats of leucine or other hydrophobic residues (L-X_6_-L-X_6_-L) that mediates dimerisation. Two bZIP monomers associate through this zipper to form dimers that preferentially recognise cis-acting elements containing an ACGT core sequence [39,40]. Plant bZIP proteins are generally classified into multiple subgroups (A–S), whose members function in hormone signalling, adaptation to environmental stresses, and regulation of secondary metabolism. In *S. miltiorrhiza*, 70 bZIP TFs have been identified [41].

NAC TFs, named after NO APICAL MERISTEM (NAM) in *Petunia hybrida* and *Arabidopsis thaliana* TRANSCRIPTION ACTIVATION FACTOR1/2 (ATAF1/2) and CUP-SHAPED COTYLEDON1/2 (CUC1/2) in *A. thaliana*, function across diverse processes, including plant development and responses to biotic and abiotic stresses. NAC proteins possess a highly conserved N-terminal NAC domain subdivided into five subdomains (A–E) that mediate DNA binding, dimer formation, and nuclear localisation. By contrast, the C-terminal region is a diversified transcriptional activation region (TAR). Some NAC proteins contain an additional C-terminal transmembrane motif and are referred to as NTLs (NAC with transmembrane motif) [42]. In *S. miltiorrhiza*, 84 NAC genes (SmNACs) have been identified [43].

TCP TFs constitute a plant-specific family characterised by a highly conserved TCP domain of approximately 58–62 amino acids. This domain forms an atypical bHLH-like structure, consisting of a stretch enriched in basic residues followed by two α-helices connected by a loop [44]. Structural analyses indicate that the TCP domain forms a β-strand at the dimer interface and uses a flexible basic loop at the N-terminus to contact DNA, establishing a distinctive three-site recognition mode. Based on structural features, TCP proteins are divided into two classes: class I (PCF type) and class II (CIN and CYC/TB1 types). Class I proteins lack four amino acids in the basic region, whereas class II proteins typically contain additional motifs such as the R domain or the ECE motif [45]. In *S. miltiorrhiza*, 84 TCP genes (SmTCPs) have been identified [46].

SPL TFs are defined by the SBP domain of approximately 76 amino acids. This domain contains two zinc-finger motifs and a nuclear localisation signal that partially overlaps with the second zinc finger, enabling specific recognition of GTAC-containing motifs in target promoters. Outside the SBP domain, SPL protein sequences show limited conservation, but distinct structural features correspond to different evolutionary groups. For example, groups I and II typically contain N-terminal phosphorylation sites that regulate protein stability under stress conditions, whereas groups III and IV possess C-terminal glutamine-rich regions that facilitate protein–protein interactions [47]. In *S. miltiorrhiza*, 15 SPL genes (SmSPLs) have been identified [48].

LBD TFs are defined by a characteristic LOB domain of approximately 100 amino acids. This domain contains a CX_2_CX_6_CX_3_C zinc-finger motif that mediates DNA binding, a central GAS region with a conserved glycine residue critical for function, and an LZL (leucine zipper-like) coiled-coil motif that supports protein dimerisation. Based on differences in the LOB domain, LBD proteins are divided into two classes: class I members possess an intact C block and LZL motif, whereas class II proteins retain the complete C block but show partial degeneration of the LZL motif. Some LBD proteins also contain a nuclear localisation signal, and their DNA-binding specificity is relatively flexible, enabling recognition of LBD motifs such as the consensus “LBDmotif” and CATTTAT-like sequences. Both homodimerisation and heterodimerisation are generally required for LBD proteins to exert regulatory activity [49]. In *S. miltiorrhiza*, 51 LBD genes (SmLBDs) have been identified [50].

### 4.2. Expression Patterns and Subcellular Localisation of Transcription Factors

Based on the 80 *S. miltiorrhiza* TFs summarised in Table 1, most genes are expressed in roots, stems, leaves, and flowers, but they show clear tissue-preferential patterns. Roots and root-associated tissues represent the most frequent expression maxima, with many genes enriched in the periderm, phloem, and xylem, where secondary metabolism is particularly active. Leaf-preferred, stem-preferred, and fibrous/lateral root–preferred expression patterns are also observed. For example, *SmMYB52*, *SmMYB111*, *SmbHLH124*, *SmbHLH148*, and *SmTGA5* are predominantly expressed in roots or root systems [51,52,53,54,55], whereas *SmHY5* and *SmbHLH130* accumulate at higher levels in leaves [56,57]. *SmMYB4*, *SmMYB39*, and *SmERF1L1* show relatively higher expression in stems [58,59]. Overall, this spatial expression landscape is consistent with the predominant accumulation of salvianolic acids in the roots of *S. miltiorrhiza*.

With respect to subcellular localisation, the majority of TFs are targeted to the nucleus. Among the reported entries, 74.4% are exclusively localised in the nucleus, and an additional 8.5% are detected mainly in the nucleus but also in other compartments, resulting in 82.9% of TFs showing nucleus-associated localisation. The remaining factors are either unreported or not clearly defined. Examples of TFs localised solely in the nucleus include *SmMYB71*, *SmTCP6*, *SmMYB98*, *SmbHLH124*, and *SmWRKY54* [9,46,53,60,61]. Dual localisation most commonly appears as “nucleus + cytoplasm” or “nucleus + plasma membrane,” as reported for *SmMYB9a*, *SmGRAS/DELLA* proteins (*SmGRAS16/19/20/21*), *SmMYB52*, and *SmbHLH53* [51,62,63,64]. This distribution is consistent with the role of TFs as transcriptional regulators. For the smaller group showing dual localisation, signal-dependent nucleo–cytoplasmic shuttling, recruitment to membrane-associated complexes, or specific protein–protein interactions may account for their subcellular patterns.

### 4.3. Hormone- and Stress-Induced Signalling in the Regulation of Salvianolic Acid Biosynthesis

AP2/ERF, bHLH, MYB, bZIP, WRKY, and GRAS TF families in *S. miltiorrhiza* show strong responsiveness to multiple elicitors, including methyl jasmonate (MeJA), JA, salicylic acid (SA), abscisic acid (ABA), and gibberellic acid (GA), and many of these factors modulate salvianolic acid biosynthesis. Overall, JA/MeJA act as the predominant upstream signals, followed by SA and ABA, whereas GA elicits detectable transcriptional responses in a subset of genes. JA/MeJA-responsive regulation is mainly associated with MYB, bHLH, WRKY, and GRAS factors, such as *SmMYB1/2/52*, *SmMYC2/2b*, and *SmWRKY14/61* [19,51,65,66,67,68]. ABA-related responses are largely mediated by bZIP and TCP proteins, including *SmbZIP1/3/38*, *SmAREB1* (identical to *SmbZIP38*), and *SmTCP17/21/19* [46,69,70,71,72]. SA- and ethylene-responsive regulation is frequently observed among WRKY and AP2/ERF members, exemplified by *SmWRKY54* and *SmERF115/2/1L1* [59,61,73,74]. In addition, GA- or GA_3_-induced responses are centred on GRAS (DELLA) proteins such as *SmGRAS16/19/20/21* [63] and are also detected in a limited number of MYB and bHLH TFs, including *SmMYB71/111* and *SmbHLH130/148/53* [52,54,57,60,64].

**Table 1:** Characteristics and regulatory information of reported transcription factor family members in *S*. *miltiorrhiza.*

TF Family	Number	Member	Regulatory Role	Inducer	Expression Pattern	Subcellular Localisation	References
MYB(R2R3)	110	SmMYB1	Positive	MeJA	leaf	Nucleus	[19]
		SmMYB2	Positive	MeJA	Root	Nucleus	[65]
		SmMYB4	Negative	MeJA, ABA, SA	Root	Nucleus	[18]
		SmMYB18	Positive	/	Root	Nucleus	[75]
		SmMYB52	Positive	MeJA, IAA	Root	Nucleus, Plasma membrane	[51]
		SmMYB111	Positive	MeJA, SA, GA	Root	Nucleus	[20,52,58,76]
		SmMYB39	Negative	MeJA	Root	Nucleus	[58,76]
		SmMYB98	Positive	/	Root	Nucleus	[9]
		SmMYB9a	Negative	/	Root	Nucleus, Cytoplasm	[62]
		SmMYB76	Negative	MeJA	Root	Nucleus	[77]
		SmMYB36	Negative	/	/	Nucleus	[78,79,80]
		SmMYB71	Negative	GA_3_	Root	Nucleus	[60]
		SmMYB97	Positive	MeJA	Root	Nucleus	[81]
		SmPAP1	Positive	MeJA, ABA, SA	leaf	/	[82]
		SmMYB37	Positive	MeJA	/	/	[83]
		SmMYB75	Positive	ABA, SA, GA, MeJA, ET, GA_3_	leaf	Nucleus	[84]
		SmMYB90	Positive	ABA, SA, GA, MeJA, ET, GA_3_	leaf	Nucleus	[84]
BHLH	127	SmMYC2	Positive	MeJA	Root	Nucleus	[20,85,86,87]
		SmbHLH3	Negative	/	Root	Nucleus	[88]
		SmbHLH92	Negative	/	Root	Nucleus	[89]
		SmJRB1	Positive	MeJA	leaf	Nucleus	[90]
		SmbHLH148	Positive	MeJA, ABA, GA	Root	Nucleus	[54]
		SmbHLH60	Negative	MeJA	Root	Nucleus	[85]
		SmbHLH59	Positive	MeJA	Root	Nucleus	[91]
		SmbHLH51	Positive	MeJA	Root	Nucleus	[52,58,76]
		SmbHLH37	Negative	MeJA	leaf	Nucleus	[86]
		SmbHLH53	-	MeJA, ABA, IAA, GA_3_	leaf	Nucleus, Plasma membrane	[64]
		SmbHLH125	-	/	Root	Nucleus	[92]
		SmbHLH7	Positive	MeJA, ABA, SA, GA	Root	Nucleus	[57]
		SmbHLH130	Positive	MeJA, ABA, GA	leaf	Nucleus	[57]
		SmMYC2b	Positive	MeJA	Root	Nucleus	[67]
		SmbHLH124	Negative	/	Stem	Nucleus	[53]
AP2/ERF	170	Sm008	/	/	Root	/	[32]
		Sm166	/	/	/	/	[32]
		SmERF115	Positive	MeJA, ET, SA	leaf	Nucleus	[73]
		SmERF2	Negative	ET, SA, MeJA	Root	Nucleus	[74]
		SmO3L3	Positive	MeJA	Root	Nucleus	[74]
		SmERF1L1	Negative	MeJA, ET, SA	leaf	Nucleus	[59]
		SmERF005	Negative	MeJA, ABA, GA	/	Nucleus	[93]
		TRINITY_DN14213_c0_g1	/	/	/	/	[94]
BZIP	70	SmbZIP1	Positive	ABA	Periderm	Nucleus	[69]
		SmbZIP2	Negative	ABA	Root	Nucleus	[95]
		SmbZIP3	Positive	ABA	/	Nucleus	[70]
		SmHY5	Positive	IAA	Root	Nucleus	[56]
		SmbZIP38	Positive	ABA	/	/	[71]
		SmTGA2	Positive	SA, GA	Root	Nucleus	[96]
		SmTGA5	Positive	SA, GA	Root	Nucleus	[55]
		SmAREB1	Positive	ABA	Root	Nucleus	[72]
		SmbZIP6	Positive	/	/	/	[97]
		SmbZIP18	Positive	/	/	/	[97]
		SmbZIP19	Positive	/	/	/	[97]
WRKY	69	SmWRKY34	Negative	ABA	Root	Nucleus	[70]
		SmWRKY61	Positive	/	Stem	/	[68]
		SmWRKY20	Positive	MeJA, ET, ABA, SA	leaf	Nucleus	[98]
		SmWRKY14	Positive	MeJA	Root	Nucleus	[99]
		SmWRKY9	-	ET, ABA, SA, MeJA	Root	Nucleus	[100]
		SmWRKY54	Negative	ET, ABA, SA, MeJA, GA_3_	leaf	Nucleus	[61]
GRAS	34	SmGRAS1	Negative	MeJA, ABA, SA, GA	Root	Nucleus	[101,102]
		SmGRAS2	Negative	MeJA, ABA, GA	Root	Nucleus	[101,102]
		SmGRAS3	Negative	MeJA, ABA, SA, GA	Root	Nucleus	[101]
		SmGRAS4	Positive	MeJA, SA, GA	Root	Nucleus	[101]
		SmGRAS5	Negative	ABA, GA	Root	Nucleus	[101]
		SmGRAS21	Positive	MeJA, GA_3_	Root	Nucleus, Cytoplasm	[63]
		SmGRAS20	Positive	MeJA, GA_3_	Root	Nucleus, Cytoplasm	[63]
		SmGRAS19	Positive	MeJA, GA_3_	Root	Nucleus, Cytoplasm	[63]
		SmGRAS16	Positive	MeJA, GA_3_	Root	Nucleus, Cytoplasm	[63]
NAC	84	SmNAC36	Positive	/	Root	Nucleus	[75]
		SmNAC1	Positive	/	/	/	[103]
		SmNAC2	Negative	/	Root	/	[104]
		SmNAC3	Negative	/	Root	/	[104]
LBD	51	LBD50	Negative	MeJA	Root	/	[50]
		LBD44	Negative	MeJA	Root	Nucleus	[105]
		LBD23	Negative	MeJA	Root	Nucleus	[106]
		LBD16	Negative	MeJA	Root	Nucleus	[106]
SPL	11	SmSPL2	Negative	/	Flower	Nucleus	[107]
		SmSPL6	Positive	MeJA, GA, ABA, IAA	leaf	Nucleus	[108]
		SmSPL7	Negative	MeJA, ABA, IAA	leaf	Nucleus	[109]
TCP	33	SmTCP17	Positive	ABA	Root	Nucleus	[46]
		SmTCP21	Positive	ABA	Root	Nucleus	[46]
		SmTCP6	Negative	ABA	Root	Nucleus	[46]
		SmTCP1	-	ABA	Root	Nucleus	[46]
		SmTCP19	-	ABA	Root	Nucleus	[46]

Beyond phytohormonal regulation, salvianolic acid biosynthesis is also responsive to diverse environmental challenges that activate distinct signalling pathways converging on a shared transcriptional network. Abiotic stresses such as UV-B irradiation induce *SmNAC1*, thereby activating upstream pathway genes and promoting phenolic acid accumulation, which is consistent with an antioxidant defence response [107]. Drought stress, largely mediated by ABA signalling, engages transcription factors including *SmWRKY34* and *SmbZIP38* to fine-tune the expression of biosynthetic genes, thereby reshaping pathway flux under water-deficit conditions [70,71]. Biotic challenges and elicitors typically activate jasmonate (JA)-associated signalling. In this context, the core JA regulator *SmMYC2*, together with interacting partners such as *SmMYB* factors, can upregulate phenolic acid biosynthetic genes, potentially strengthening chemical defence capacity [19,87]. This induction is further modulated by repressor proteins such as SmJAZ1 and SmJAZ8, illustrating how stress cues are gated and integrated at the transcriptional level [58,90]. Collectively, these examples indicate that the salvianolic acid regulatory network functions as an integrative hub, translating both abiotic and biotic-associated stimuli into tailored adjustments of phenolic metabolism, enabling dynamic optimisation of specialised metabolite profiles under fluctuating environments.

## 5. Transcriptional Regulation of Salvianolic Acids by Transcription Factors

The transcriptional regulation of salvianolic acid biosynthesis involves multiple TF families, within which individual members can function as activators, repressors, or context-dependent regulators. This functional divergence often reflects evolutionary specialisation into distinct clades or subfamilies. Mechanistically, it is shaped by differences in transcriptional complex assembly, target gene selection across key enzymatic nodes, and tissue- or condition-specific expression. The following sections discuss the major TF families using this integrative framework, with an emphasis on positive regulation, negative regulation, and complex-mediated regulation. This mechanistic understanding is essential for the rational selection and stacking of TFs to achieve predictable metabolic engineering outcomes.

Unless otherwise specified, functional evidence for the TFs summarised below was mainly obtained from *S. miltiorrhiza* hairy-root cultures and/or root tissues, where phenolic acids are typically enriched; TF–target relationships were further supported by promoter-binding and transient expression assays. Information on tissue/organ expression patterns is summarised in Table 1.

### 5.1. Regulatory Roles of MYB Transcription Factors in Salvianolic Acid Biosynthesis

Several R2R3-MYB TFs in *S. miltiorrhiza* function as transcriptional activators that directly upregulate key enzymes in both the phenylpropanoid and tyrosine-derived branches, thereby significantly enhancing salvianolic acid accumulation. *SmMYB1* is induced by MeJA and activates multiple pathway genes, including *PAL1*, *C4H1*, *4CL1*, *TAT1*, *HPPR1*, *RAS1*, and *CYP98A14*, which results in increased salvianolic acid production. In *SmMYB1*-overexpressing hairy roots, total salvianolic acids reach approximately twice the level of the control, whereas expression of a dominant repressor version (*SmMYB1*-SRDX) reduces the content to 57.3% of the control [19]. Overexpression of *SmPAP1*, a homologue of *AtPAP1*, also strongly stimulates the pathway. Transgenic plants exhibit 1.52–1.95-fold higher RA and 1.39–1.88-fold higher Sal B than the wild type, accompanied by upregulation of *PAL*, *C4H*, *4CL*, and other structural genes, which indicates strong activation of the phenylpropanoid route [82]. In transgenic hairy roots, *SmMYB2* overexpression increases total phenolic acids to nearly threefold that of the control, whereas suppression of *SmMYB2* results in reduced levels [65]. *SmMYB18* primarily targets the tyrosine-derived branch. In overexpression lines, *SmTAT2* transcripts increase by 2.16–12.58-fold, with RA and Sal B levels elevated by 2.06–2.68-fold and 1.64–2.36-fold, respectively, whereas knockout of *SmMYB18* causes significant decreases in these metabolites [75].

Consistent pro-accumulation effects are also reported for *SmMYB52*, *SmMYB97*, *SmMYB98*, *SmMYB75*, *SmMYB90*, and *SmMYB111*. In *SmMYB52*-overexpressing lines, Sal B increases by 2.1–2.2-fold, and both total phenolics and RA are significantly elevated, whereas RNAi-mediated silencing reduces Sal B by 4.5–5.5-fold [51]. *SmMYB97* overexpression results in 1.6–2.0-fold higher RA and 1.3–2.5-fold higher Sal B [81], and *SmMYB98* overexpression results in up to 1.5-fold increases in total RA + Sal B, which decline substantially in knockout backgrounds [9]. *SmMYB75* and *SmMYB90* are MeJA-responsive R2R3-MYB factors that positively regulate salvianolic acid accumulation. Overexpression of either TF enhances expression of key pathway genes such as *Sm4CL1*, *SmTAT*, *SmRAS*, and *SmCYP98A14*, whereas repression of *SmMYB75* or *SmMYB90* in hairy roots significantly decreases total salvianolic acid content [84]. *SmMYB37* is a target of miR396b and binds to the promoter of the phenylpropanoid gene C4H. Overexpression of miR396b markedly reduces *SmMYB37* transcript levels, and total phenolic acids decline to 72.1% of the control, which suggests a negative correlation and implies that elevated *SmMYB37* expression promotes phenolic acid accumulation [83]. Among these activators, *SmMYB111* shows a strong effect. In *SmMYB111*-overexpressing hairy roots, RA content increases by approximately 2.19-fold, and Sal B reaches up to 4.65-fold of the wild-type level [20,52,58].

Substantial evidence also indicates that several MYB TFs act as negative regulators of salvianolic acid biosynthesis. *SmMYB4* binds to the promoters of *SmPAL*, *SmC4H*, and *SmTAT1* and represses their transcription. In *SmMYB4*-overexpressing plants, RA and Sal B contents decrease to 56.6% and 57.6% of wild-type levels, respectively, whereas in RNAi lines total phenolic acids increase to approximately 1.8-fold of the wild type [18]. When CA, RA, and Sal B were quantified in *SmMYB9a*-overexpressing and -silenced hairy roots, little change was detected in overexpression lines, whereas in RNAi lines CA, RA, and Sal B increase to 1.9-, 1.47-, and 2.4-fold of wild-type levels, respectively [62]. Overexpression of *SmMYB76* reduces total phenolic acid levels by 39–69%, whereas knockout of *SmMYB76* results in a 47–132% increase compared with the control [77]. Similarly, loss of *SmMYB71* function raises RA and Sal B contents to 1.5- and 2-fold of wild-type levels, respectively, whereas *SmMYB71* overexpression reduces RA to 30% and Sal B to 60% of the wild type, which demonstrates a strong negative regulatory role [60]. *SmMYB36* represses transcription of the key phenolic acid biosynthetic genes *SmRAS* and *SmGAPC* by directly binding to MYB recognition elements in their promoters and simultaneously downregulates the upstream TF *SmERF115*, thereby restricting accumulation of downstream products. In *SmMYB36*-overexpressing hairy roots, RA and Sal B contents decrease by more than 50%, whereas in *SmMYB36*-SRDX dominant-interference lines both metabolites increase by more than 1.5-fold, consistent with the observed gene expression changes [78,79].

*SmMYB39* not only represses multiple pathway genes but also disrupts assembly and activity of the MYB–bHLH–WD40 regulatory complex. *SmMYB39* interacts with all components of the *SmMYB111*–*SmbHLH51*–*SmTTG1* complex, which blocks complex formation, and directly suppresses transcriptional activation of *SmC4H1* and other targets by *SmbHLH51*. Therefore, *SmMYB39* exerts negative regulation at both transcriptional and protein–protein interaction levels. In *SmMYB39*-overexpressing hairy roots, RA and Sal B contents decline to approximately 5% and 22.6% of wild-type levels, respectively, whereas RNAi-mediated silencing increases RA and Sal B to approximately 3.81- and 4.23-fold of the wild type. *SmMYB39* also interacts with the jasmonate signalling repressor *SmJAZ1*, forming a higher-order negative regulatory module within the JA–TF network [58].

*SmMYB111* does not function alone but cooperates with *SmbHLH51* and *SmTTG1* to form an MYB–bHLH–WD40 (MBW) ternary complex that strongly enhances transcriptional activation of target genes [52,110]. Activity of *SmMYB111* is further regulated at multiple levels. The microRNA Smi-miR858a targets *SmMYB111* transcripts for cleavage, which reduces its expression and downregulates downstream pathway genes, leading to decreased phenolic acid accumulation. In Smi-miR858a-overexpressing plants, Sal B content is reduced by 87.2% relative to the wild type [111]. *SmMYB76* interacts with the jasmonate signalling repressor *SmJAZ9*, and together they strengthen transcriptional repression of downstream genes. *SmJAZ9* promotes *SmMYB76* expression and enhances its repressive activity, whereas MeJA treatment induces *SmJAZ9* degradation, which alleviates *SmMYB76*-mediated repression of *SmPAL*, *Sm4CL2*, and *SmRAS1* and releases inhibition of phenolic acid biosynthesis [77]. *SmMYB36* interacts with *SmCSN5*, which stabilises *SmMYB36* by inhibiting ubiquitin-mediated degradation and thereby reinforces its suppressive effect on phenolic acid production [80]. When *SmMYB36* is fused to the strong activation domain VP16 to generate *SmMYB36*–VP16, its ability to activate *SmRAS* is greatly enhanced, and RA content reaches 5.05-fold that of the control [112]. *SmMYB1* can also form a complex with *SmMYC2*. Dual-luciferase assays show that combined action of *SmMYB1* and *SmMYC2* activates *CYP98A14* more strongly than *SmMYB1* alone, which indicates that their cooperation substantially increases phenolic acid biosynthetic activity [19].

### 5.2. Regulatory Roles of bHLH Transcription Factors in Salvianolic Acid Biosynthesis

bHLH TFs participate in both positive and negative regulation of phenolic acid biosynthesis in *S. miltiorrhiza*. On the positive side, *SmMYC2/SmMYC2b*, *SmJRB1*, *SmbHLH59*, *SmbHLH51*, *SmbHLH7*, *SmbHLH130*, and *SmbHLH148* promote salvianolic acid accumulation. *SmMYC2* is strongly induced by MeJA and binds E/G-box elements in target promoters to activate key enzymes such as *SmPAL*, *SmTAT1*, and *SmCYP98A14*, thereby enhancing synthesis of RA, Sal B, and related metabolites. In *SmMYC2*-overexpressing plants, root Sal B content increases by approximately 1.88-fold relative to the control [85,87], whereas RNAi knockdown of *SmMYC2b* reduces RA and Sal B to 0.09- and 0.05-fold of control levels, respectively [67]. *SmJRB1* is rapidly induced by MeJA, directly targets a G-box in the *RAS1* promoter, and coordinately upregulates *RAS1*, *CYP98A14*, and multiple upstream genes, including *PAL*, *4CL*, *TAT*, and *HPPR*, which promotes RA and Sal B accumulation [90]. *SmbHLH59* binds promoter elements in both the phenylalanine- and tyrosine-derived precursor branches and at their convergence node to enhance transcriptional activity; its function is repressed by *SmJAZ1* and *SmJAZ8*, representing a canonical MeJA–JAZ–bHLH regulatory module [91]. *SmbHLH51* increases RA and Sal B production by directly or indirectly activating key pathway genes and forms an MBW complex with *SmMYB111* and *SmTTG1* that further amplifies transcriptional output [76]. In addition, *SmbHLH7* and *SmbHLH130* bind G-box motifs in the promoters of *C4H1*, *TAT*, *HPPR*, and *CYP98A14* to activate transcription, leading to significant increases in RA and Sal B levels in overexpressing hairy roots [57]. *SmbHLH148* is strongly induced by ABA, and its overexpression causes sustained upregulation of most genes in both the phenolic acid and tanshinone pathways. In the OEbHLH148-3 line, Sal B content reaches approximately 5.99-fold that of the control [54].

Several bHLH TFs, including *SmbHLH3*, *SmbHLH60*, *SmbHLH92*, *SmbHLH37*, and *SmbHLH124*, act as negative regulators of phenolic acid biosynthesis. *SmbHLH3* directly binds G-box motifs in the *SmTAT* and *SmHPPR* promoters to repress transcription; in *SmbHLH3*-overexpressing lines, RA and Sal B levels decrease to approximately 50% and 38% of the control, respectively [88]. Overexpression of *SmbHLH60* similarly reduces phenolic acid accumulation, whereas CRISPR/Cas9 knockout of *SmbHLH60* results in a marked increase in phenolic acids. Notably, *SmbHLH60* transcript levels are rapidly downregulated after MeJA treatment, consistent with a negative feedback mechanism that attenuates excessive JA signalling [85]. RNAi-mediated suppression of *SmbHLH92* upregulates *PAL1*, *CYP98A14*, and other pathway genes and increases phenolic acid accumulation, indicating a repressive role for *SmbHLH92* [89]. *SmbHLH37* binds the promoters of *SmTAT1* and *SmPAL1* and counteracts the activating effect of *SmMYC2*; in *SmbHLH37*-overexpressing material, Sal B is reduced by approximately 1.6–1.8-fold and RA by approximately 1.7–2.0-fold, accompanied by broad downregulation of phenolic acid pathway genes [86]. Overexpression of *SmbHLH124* causes coordinated repression of multiple pathway nodes, including *PAL3*, *4CL3/4/9*, *RAS1*, and *HPPR3*, which leads to substantial decreases in RA and Sal B [53].

Some bHLH members display context-dependent or bidirectional effects. For example, *SmbHLH53* represses *SmTAT1*, *SmPAL1*, and *SmC4H1* while activating *Sm4CL9*. In the corresponding transgenic lines, opposing changes in upstream and downstream genes largely offset each other, resulting in no significant difference in total RA and Sal B content relative to the control [64]. *SmbHLH125* binds the *Sm4CL3* promoter and negatively regulates its expression. Gene editing of *SmbHLH125* produces a differential metabolic response, with decreased RA and increased Sal B levels, which suggests branch-selective control over metabolic flux partitioning [92].

### 5.3. Regulatory Roles of ERF Transcription Factors in Salvianolic Acid Biosynthesis

*SmERF115* is a jasmonate-responsive TF. Its overexpression in *S. miltiorrhiza* hairy roots significantly upregulates *SmRAS1*, accompanied by increased expression of *PAL3*, *4CL5*, *TAT3*, and *RAS4*, and results in an average increase of approximately 1.43-fold in Sal B, whereas RA and Sal A show no significant changes. Conversely, in *SmERF115*-RNAi lines, Sal B levels decline to approximately 50% of those in the control [73]. Another ERF member, *SmO3L3*, a close homologue of *A. thaliana* ERF113, similarly enhances phenolic acid accumulation when overexpressed in hairy roots, with the best line reaching 1.86-fold higher total salvianolic acids than the control. In *SmO3L3* RNAi lines, salvianolic acid content averages 73% of control levels [74].

Some AP2/ERF members in *S. miltiorrhiza* function as repressors of phenolic acid accumulation through direct inhibition of key biosynthetic genes or competition for metabolic flux. *SmERF005* is responsive to both ABA and MeJA. Under 50 μM ABA treatment, its transcript level declines rapidly, reaching a minimum of 5.79% at 12 h. Functional assays show that *SmERF005* binds the GCC-box element in the *SmCYP98A14* promoter and reduces promoter activity to 29% of the control in dual-luciferase assays. Because biomass of *SmERF005*-overexpressing lines was insufficient, metabolite levels were not quantified; however, in *SmERF005* RNAi hairy roots, total phenolic acids increase by approximately 28% relative to the control [93]. *SmERF1L1* is a JA-sensitive AP2/ERF factor that is rapidly induced by MeJA and related elicitors. Overexpression of *SmERF1L1* in hairy roots reduces total phenolic acids to approximately 66% of control levels, with significant decreases in RA and Sal A. Most core phenolic acid biosynthetic genes show no obvious transcriptional changes, whereas *SmHPPD* is differentially expressed, consistent with its position at a pathway branch point [59]. *SmERF2* also acts as a negative regulator of salvianolic acid biosynthesis. Overexpression reduces total salvianolic acids to approximately 66% of control levels, whereas *SmERF2* RNAi lines show contents comparable to the control, likely reflecting functional redundancy among AP2/ERF members [74].

Reports describing ERF TFs that directly regulate salvianolic acid biosynthesis in *S. miltiorrhiza* remain limited. Based on available expression profiles and functional assays, three additional TFs have been proposed as candidates in this pathway. quantitative reverse transcription PCR (qRT–PCR) analysis indicates that *Sm008* and *Sm166* show highest expression in the phloem and xylem of roots, with tissue-specific patterns closely matching that of *SmRAS1*. They also display strong co-expression with *SmRAS*, which suggests a positive association with salvianolic acid biosynthesis [32]. In contrast, the drought-induced DREB-type factor *TRINITY_DN14213_c0_g1* has direct functional support. Dual-luciferase assays show significant transactivation of the *SmC4H* and *SmRAS* promoters, indicating that this factor may act as a positive regulator of salvianolic acid production [94].

### 5.4. Regulatory Roles of WRKY Transcription Factors in Salvianolic Acid Biosynthesis

*SmWRKY40*, identified from a Sichuan ecotype of *S. miltiorrhiza*, is a core component of the phenolic acid biosynthetic gene cluster and functions as an important positive regulator of salvianolic acid biosynthesis. Overexpression of *SmWRKY40* in hairy roots significantly increases RA and Sal B levels, accompanied by coordinated upregulation of key pathway genes, including *SmTAT2*, *SmHPPR*, *SmRAS*, and *SmCYP98A14*. Conversely, CRISPR/Cas9-mediated knockout of *SmWRKY40* causes marked reductions in RA and Sal B, together with inhibited root growth and softened root texture. This regulatory function appears conserved across species. In *A. thaliana*, *SmWRKY40* overexpression or complementation of the wrky40 mutant promotes accumulation of phenolic acid–related metabolites [113].

*SmWRKY20* was identified from YE/Ag^+^-treated transcriptomes as another WRKY factor regulating salvianolic acid biosynthesis. In *SmWRKY20*-overexpressing lines, Sal B increases by 13–40% and total salvianolic acids by 16–40%, whereas in RNAi lines these values decrease by 15–40% and 8–37%, respectively, which indicates a positive regulatory role. *SmWRKY20* shows no autoactivation activity in yeast, which suggests that cofactors may be required in planta to achieve full transcriptional activity [98]. Jasmonate signalling studies further show that *SmWRKY14* directly binds and activates the promoters of *SmPAL1*, *SmC4H1*, *SmTAT1*, *SmHPPR*, and *SmRAS1*, which leads to substantial increases in phenolic acid content. Overexpression of *SmWRKY14* in hairy roots raises total phenolic acids and Sal B to approximately 1.46-fold and 2.99-fold of control levels, respectively. *SmWRKY14* also participates in jasmonate signalling through interaction with JAZ proteins, forming a hormone-responsive regulatory node that connects defence signalling with secondary metabolism [99]. Although *SmWRKY61* was initially characterised for its role in tanshinone biosynthesis, its overexpression in hairy roots also causes a pronounced increase in RA to 3.33-fold of control levels, whereas Sal B and CA remain unchanged. Consistently, RA-branch genes such as *Sm4CL3*, *SmTAT1*, and *SmCYP98A14* are upregulated, which indicates that *SmWRKY61* preferentially promotes the RA branch of the phenolic acid pathway [68].

In contrast, *SmWRKY34*, a subgroup IIa WRKY TF induced by ABA, functions as a strong repressor of phenolic acid accumulation. Overexpression of *SmWRKY34* reduces total phenolic acids to an average of 22.7% of control levels, with some lines declining to 6.3%, whereas CRISPR/Cas9-mediated knockout increases total phenolic acids to approximately 1.5-fold of the control [70]. *SmWRKY54* also acts as a negative regulator. Its overexpression in hairy roots decreases total salvianolic acids to 29.1% of control levels, while RNAi-mediated suppression elevates them to approximately 1.2-fold relative to the control [61]. Mechanistic evidence suggests that *SmWRKY54* redirects metabolic flux away from phenolic acid biosynthesis by downregulating *Sm4CL1* and upregulating *SmHPPD*, which limits salvianolic acid accumulation.

*SmWRKY9* shows a more complex regulatory profile. Overexpression of *SmWRKY9* increases RA content to 2.22-fold of control levels, whereas expression of a dominant repressor (*SmWRKY9*-SRDX) markedly reduces RA. *SmWRKY9* directly binds to and activates the promoters of *SmRAS1* and *SmCYP98A14* and also upregulates *SmPAL3* and *Sm4CL2/3/8*, thereby promoting RA biosynthesis. Notably, *SmWRKY9* shows a negative association with Sal B. Both overexpression and SRDX lines exhibit lower Sal B levels than the control, which suggests that *SmWRKY9* may restrict or redirect metabolic flux at the step converting RA to Sal B [100].

### 5.5. Regulatory Roles of bZIP Transcription Factors in Salvianolic Acid Biosynthesis

In *S. miltiorrhiza*, several bZIP TFs positively regulate salvianolic acid biosynthesis and often function as integrative nodes that connect hormone and light signalling with pathway gene expression. Overexpression of *SmbZIP1* in hairy roots increases *C4H1* transcript levels by approximately 3.5-fold and *HPPR* by approximately 2.5-fold, which results in a substantial rise in total phenolic acids. In the strongest line (OE-4), total salvianolic acids reach approximately 4.1-fold of the control, whereas CRISPR/Cas9 knockout of *SmbZIP1* significantly decreases Sal B, with the lowest line retaining only 53.1% of control levels [69]. Within the SA signalling pathway, *SmTGA2* shows strong positive effects on salvianolic acid production. In *SmTGA2*-overexpressing hairy roots, RA and Sal B reach 2.02- and 1.29-fold of wild-type levels, respectively, whereas antisense suppression reduces them to 0.82- and 0.56-fold of the wild type. At the mechanistic level, *SmTGA2* upregulates *SmPAL1*, *SmTAT1*, *SmRAS1*, *SmCYP98A14*, *SmC4H1*, and *SmHPPR1*, with *SmCYP98A14* showing the strongest induction (3.45–9.56-fold). *SmTGA2* also interacts specifically with the SA receptor *SmNPR1*, forming an SA–*SmNPR1*–*SmTGA2* module that promotes salvianolic acid biosynthesis [96].

The light signalling factor *SmHY5* also positively regulates phenolic acid accumulation. In *SmHY5*-overexpressing plants, RA and Sal B increase significantly, reaching up to 1.81- and 1.39-fold of wild-type levels, respectively. In RNAi lines, both metabolites decrease markedly. Notably, Sal A in the RNAi-18 line increases to 2.67-fold of the wild type, which suggests redistribution of metabolic flux through alternative branches or bypass routes [56]. As an ABA-responsive bZIP, *SmAREB1* forms homodimers and binds ABRE cis-elements in the *SmTAT* promoter to directly activate transcription, while also upregulating *SmRAS*, *SmPAL1*, and *Sm4CL1*. These coordinated changes increase channelling of precursors towards salvianolic acids. In *SmAREB1*-overexpressing hairy roots, RA, Sal B, and total salvianolic acids reach 1.16-, 1.22-, and 1.19-fold of control levels, respectively, whereas in RNAi lines they decline to 71%, 86%, and 75% of the control [71]. Another family member, *SmbZIP3*, directly recognises two ABRE motifs in the *SmTAT* promoter and activates this gene. In *SmbZIP3*-overexpressing lines, PAL and TAT transcripts increase, whereas RAS and CYP98A14 decrease; nevertheless, overall metabolic flux shifts towards RA and Sal B, and total phenolic acids average approximately 1.59-fold of the control. These results indicate that *SmbZIP3* enhances early steps in the tyrosine-derived branch to increase precursor supply despite negative feedback at later modification steps [69]. At the level of hormone–kinase–TF crosstalk, *SmSnRK2.6* and *SmAREB1* form an ABA-activated regulatory module. Overexpression of either component increases RA and Sal B levels, which highlights a tightly coupled ABA–SnRK2–bZIP cascade that promotes salvianolic acid biosynthesis [72].

Among bZIP TFs that repress salvianolic acid biosynthesis, *SmbZIP2* has the most direct experimental support. In *SmbZIP2*-overexpressing hairy roots, total salvianolic acids average only 54% of the empty-vector control, whereas CRISPR/Cas9-induced mutations in *SmbZIP2* increase salvianolic acid levels by 23–53% relative to the control. Mechanistic analyses show that *SmbZIP2* represses transcription of PAL, a key rate-limiting enzyme in the pathway [95].

Some bZIP TFs remodel salvianolic acid flux through more complex, indirect mechanisms that include signalling integration, protein–protein interactions, and competition between metabolic branches. First, within the SA pathway, *SmTGA5* activates *SmTAT1* and promotes salvianolic acid accumulation, but the *SmNPR4*–*SmTGA5* module imposes an upper limit on this response under SA induction. In *SmNPR4*-overexpressing lines, Sal B levels decrease to 46–56% of the control, whereas in *SmNPR4*-RNAi lines the SA-induced increases in RA and Sal B reach 1.19- and 1.23-fold of the control, respectively. These findings indicate that receptor–TF pairing provides threshold control over SA-mediated stimulation of phenolic acid biosynthesis [55]. Second, *SmbZIP1* displays an antagonistic regulatory pattern across the two major secondary metabolic pathways. It upregulates *C4H1* and *HPPR*, which strongly increases salvianolic acid accumulation, while binding the G-box in the geranylgeranyl diphosphate synthase (*GGPPS*) promoter to repress its transcription. This repression results in up to 95.3% reduction in total tanshinones in overexpression lines, with significant increases observed in knockout lines. These results indicate that *SmbZIP1* contributes to metabolic partitioning between competing branches and indirectly favours synthesis of water-soluble salvianolic acids [69].

Third, PAL, a pivotal enzyme in the phenolic acid pathway, is transcriptionally repressed by *SmbZIP2*. *SmbZIP2* physically interacts with *SmSnRK2.6*, which suggests that *SmSnRK2.6* may modulate *SmbZIP2* activity through phosphorylation [95]. In addition, several other bZIP proteins, including bZIP6, bZIP18, and bZIP19, bind promoters of pathway genes, although their metabolic roles remain incompletely defined. Yeast one-hybrid and electrophoretic mobility shift assay (EMSA) assays show specific binding of bZIP18 to the *Sm4CL2* promoter and of bZIP6 and bZIP19 to the *SmTAT* promoter, which suggests that these factors may influence phenolic acid biosynthesis by acting at key regulatory nodes such as 4CL and TAT [97].

### 5.6. Regulatory Roles of GRAS Transcription Factors in Salvianolic Acid Biosynthesis

Overexpression of *SmGRAS4* significantly upregulates key genes in the salvianolic acid biosynthetic pathway, including *SmC4H1*, *Sm4CL1*, and *SmCYP98A14*, which leads to increased phenolic acid accumulation. In *SmGRAS4*-overexpressing hairy roots, RA reaches 1.19–1.58-fold and Sal B 1.13–1.29-fold of the empty-vector control, whereas antisense lines show reduced levels of both metabolites. In addition, interference lines targeting the four members of the *SmDELLA* subfamily (*SmDELLA1–4*) all show lower total phenolic acid contents than the control, with *SmDELLA1* and *SmDELLA3* exhibiting particularly strong reductions to 34% and 37% of control levels, respectively. These results indicate that *SmDELLA* genes act as positive regulators of total phenolic acid accumulation in *S. miltiorrhiza* [63].

In contrast, *SmGRAS1*, *SmGRAS2*, *SmGRAS3*, and *SmGRAS5* function as negative regulators of phenolic acid biosynthesis in *S. miltiorrhiza*. Overexpression of these TFs markedly downregulates expression of key genes in the salvianolic acid pathway, which results in pronounced decreases in metabolite accumulation. RA levels decline to 0.68–0.83-fold and Sal B to 0.42–0.89-fold of the empty-vector control. In antisense lines, both metabolites increase significantly. For example, RA in *SmGRAS3* antisense lines reaches up to 1.63-fold of the control, which further supports the repressive roles of these GRAS factors in phenolic acid production [101,102].

### 5.7. Regulatory Roles of Other Transcription Factor Families in Salvianolic Acid Biosynthesis

In addition to the TF families described above, several regulators of salvianolic acid biosynthesis belong to the NAC, LBD, SPL, and TCP families.

*SmNAC36* binds ABRE elements in the promoters of *SmMYB18* and *SmTAT2* and activates their transcription. *SmMYB18* subsequently recognises an MBS motif in the *SmTAT2* promoter and enhances its transcription, which increases activity of the tyrosine-branch enzyme TAT2 and markedly elevates RA and Sal B accumulation. In *SmNAC36*-overexpressing hairy roots, RA increases by 2.51–3.66-fold and Sal B by 2.72–7.02-fold relative to the control [75]. ultraviolet B (UV-B)–induced *SmNAC1* acts as an upstream regulator that directly binds and activates CATGTG/CATGTC motifs in the *PAL3* and *TAT3* promoters. This activation upregulates *PAL3*, *TAT3*, and *HPPR3*, enhances PAL and TAT enzyme activities, and results in significant increases in RA and Sal B in overexpression lines, with corresponding decreases in RNAi lines [103]. Overexpression of *SmNAC2* significantly elevates RA content, whereas its silencing markedly increases Sal B to 2.43–3.21-fold of wild-type levels. At the transcriptional level, *SmNAC2* overexpression upregulates upstream *4CL* and *TAT* transcripts, whereas RNAi lines show further induction of *PAL1*, *4CL*, *TAT*, and *CYP98A14*. By contrast, *SmNAC3* overexpression reduces both RA and Sal B, whereas *SmNAC3* silencing increases their levels. *SmNAC3* primarily affects *C4H* expression, and its RNAi also upregulates *PAL2*, which indicates that *SmNAC3* represses the upstream phenylpropanoid pathway [104].

*SmLBD50* overexpression markedly reduces total phenolic acids and RA in *S. miltiorrhiza*. Mechanistically, *SmLBD50* downregulates multiple genes in both the phenylpropanoid branch (*PAL1/3*, *C4H1*, *4CL2/3*) and the tyrosine-derived branch (*TAT1/3*, *HPPR1*), and simultaneously represses downstream *SmRAS* and *CYP98A14*, thereby inhibiting phenolic acid biosynthesis. In *SmLBD23*-overexpressing lines, root RA content decreases to 21–51% of the control, whereas in *SmLBD16* RNAi roots RA levels increase to 1.46–1.91-fold of the control [106]. *SmLBD44* also acts as a negative regulator of total phenolic acid accumulation. In overexpression lines, total phenolic acids decrease to approximately 52–84% of control levels, whereas in RNAi lines they increase to 1.28–1.51-fold. Bimolecular fluorescence complementation assays show that *SmLBD44* interacts with *SmJAZ1*, and transient transcription assays indicate that *SmJAZ1* abolishes *SmLBD44*-mediated repression of the *SmKSL1* promoter. These findings indicate that JAZ–LBD interactions modulate the balance between competing specialised metabolic pathways [105].

*SmSPL2* represses salvianolic acid biosynthesis by downregulating multiple pathway genes across different branches, including *SmTAT1*, *SmPAL*, *Sm4CL9*, *SmC4H1*, and *SmRAS2*. In *SmSPL2*-overexpressing plants, RA and Sal B decrease markedly, with root RA reaching 59–78% and Sal B 20–36% of control levels [107]. By contrast, *SmSPL6* functions as a positive regulator. In *SmSPL6*-overexpressing lines, both RA and Sal B increase significantly, and root Sal B reaches 5.18–8.19-fold of the control [108]. *SmSPL7* shows an intermediate pattern and specifically affects the downstream branch towards Sal B. In *SmSPL7*-overexpressing lines, root Sal B decreases to 35–65% of control levels, whereas RA remains largely unchanged, which suggests selective regulation of flux at the RA-to-Sal B conversion step [109].

Under ABA induction, TCP family members display site-specific binding and context-dependent regulation of the promoters of the branch-specific genes *SmRAS1* and *SmCYP98A14* in the salvianolic acid pathway. *SmTCP17* and *SmTCP21* tend to activate both *CYP98A14* and *RAS1*, whereas *SmTCP6* represses *RAS1*. *SmTCP1* and *SmTCP19* activate *CYP98A14* but repress *RAS1*, which indicates that their net effects on salvianolic acid biosynthesis depend on expression context and protein abundance. Because in planta binding and metabolic phenotypes remain incompletely resolved, these TCP factors are currently regarded as candidate regulators of salvianolic acid biosynthesis [46].

### 5.8. Core Regulatory Hubs and Design Principles for Metabolic Engineering

Current evidence indicates that TF-mediated control of salvianolic acid biosynthesis is organised around a limited number of recurring regulatory hubs rather than isolated single-factor effects. A JA-gated hub centred on *SmMYC2* provides a switch-like mechanism in which JAZ repressors constrain transcriptional activation under basal conditions and JA-associated cues relieve repression to enable coordinated induction of biosynthetic genes. A second hub involves cooperative and antagonistic TF complexes, in which MYB–bHLH partnerships and related multi-protein assemblies shape transcriptional output through partner availability, competitive binding, and complex reconfiguration, thereby producing context-dependent activation or attenuation. A third hub reflects convergent regulation of bottleneck enzymatic nodes, with repeated TF inputs targeting *PAL*/*C4H*/*4CL* in the phenylpropanoid branch and *TAT*/*HPPR*/*RAS*/*CYP98A14* in the tyrosine-derived and downstream steps, indicating that flux control is frequently executed by jointly tuning a small set of rate-limiting reactions. These hubs provide practical design principles for precision metabolic engineering, including prioritising TF combinations that coordinately control multiple bottleneck enzymes, selecting regulators that participate in cooperative complexes, and stacking activators with appropriate de-repression or feedback-gating elements to achieve predictable and stable flux rewiring.

## 6. Conclusions and Perspectives

This review synthesises evidence that the transcriptional regulation of salvianolic acid biosynthesis in *S. miltiorrhiza* is orchestrated through a limited set of core regulatory hubs rather than by isolated transcription factors. These hubs—primarily the jasmonate-gated switch centred on SmMYC2, the cooperative MYB-bHLH-WD40 complexes, and the multifactor convergent control of bottleneck enzymatic steps—collectively integrate hormonal and environmental signals to fine-tune pathway flux with spatial and temporal precision.

Elucidating these hubs shifts the focus from cataloguing individual regulators to establishing a mechanistic blueprint for rational metabolic engineering. Key design principles emerge from this blueprint, including the stacking of transcription factors that co-target multiple rate-limiting enzymes, the exploitation of cooperative transcription factor complexes for synergistic activation, and the coupling of inducible derepression mechanisms with strong activators to create dynamic, high-yield production systems. Implementing these principles in hairy-root cultures, whole-plant breeding, or synthetic microbial chassis offers a direct route to achieving stable, high-level salvianolic acid production.

Looking forward, translating this blueprint into robust breeding and bioproduction strategies will require overcoming persistent challenges. Key among these are decoding the crosstalk between regulatory hubs, resolving cell-type-specific expression patterns of hub components through spatial and single-cell omics, and balancing engineered metabolic flux with plant growth and development. Integrating the described regulatory logic with emerging technologies—such as CRISPR-based genome editing for precise hub manipulation, synthetic promoter design for context-specific expression, and computational approaches for predicting optimal transcription factor combinations—will enable the next generation of predictive, design-driven improvement of *S. miltiorrhiza* and other valuable medicinal plants.

## Data Availability

Data are contained within the article.
